# High ApoB/ApoA-I Ratio Predicts Post-Stroke Cognitive Impairment in Acute Ischemic Stroke Patients with Large Artery Atherosclerosis

**DOI:** 10.3390/nu15214670

**Published:** 2023-11-04

**Authors:** Minwoo Lee, Jae-Sung Lim, Yerim Kim, Soo Hyun Park, Sang-Hwa Lee, Chulho Kim, Byung-Chul Lee, Kyung-Ho Yu, Jae-Jun Lee, Mi Sun Oh

**Affiliations:** 1 Department of Neurology, Hallym University Sacred Heart Hospital, Hallym Neurological Institute, Hallym University College of Medicine, Anyang 14068, Republic of Korea; minwoo.lee.md@gmail.com (M.L.); ssbrain@hallym.or.kr (B.-C.L.); ykh1030@hallym.or.kr (K.-H.Y.); 2 Institute of New Frontier Research Team, Hallym University College of Medicine, Chuncheon 24252, Republic of Korea; iloveu59@hallym.or.kr; 3 Department of Neurology, Asan Medical Center, Ulsan University College of Medicine, Seoul 05505, Republic of Korea; jaesunglim@amc.seoul.kr; 4 Department of Neurology, Kangdong Sacred Heart Hospital, Hallym University College of Medicine, Seoul 24252, Republic of Korea; brainyrk@gmail.com (Y.K.); g2skhome@gmail.com (S.H.P.); 5 Department of Neurology, Chuncheon Sacred Heart Hospital, Hallym University College of Medicine, Chuncheon 24252, Republic of Korea; bleulsh@naver.com (S.-H.L.); gumdol52@naver.com (C.K.)

**Keywords:** stroke, dementia, apolipoprotein, atherosclerosis

## Abstract

Background: We aimed to investigate the association between the ApoB/ApoA-I ratio and post-stroke cognitive impairment (PSCI) in patients with acute stroke of large artery atherosclerosis etiology. Methods: Prospective stroke registry data were used to consecutively enroll patients with acute ischemic stroke due to large artery atherosclerosis. Cognitive function assessments were conducted 3 to 6 months after stroke. PSCI was defined as a z-score of less than −2 standard deviations from age, sex, and education-adjusted means in at least one cognitive domain. The ApoB/ApoA-I ratio was calculated, and patients were categorized into five groups according to quintiles of the ratio. Logistic regression analyses were performed to assess the association between quintiles of the ApoB/ApoA-I ratio and PSCI. Results: A total of 263 patients were included, with a mean age of 65.9 ± 11.6 years. The median NIHSS score and ApoB/ApoA-I ratio upon admission were 2 (IQR, 1–5) and 0.81 (IQR, 0.76–0.88), respectively. PSCI was observed in 91 (34.6%) patients. The highest quintile (Q5) of the ApoB/ApoA-I ratio was a significant predictor of PSCI compared to the lowest quintile (Q1) (adjusted OR, 3.16; 95% CI, 1.19–8.41; *p*-value = 0.021) after adjusting for relevant confounders. Patients in the Q5 group exhibited significantly worse performance in the frontal domain. Conclusions: The ApoB/ApoA-I ratio in the acute stage of stroke independently predicted the development of PSCI at 3–6 months after stroke due to large artery atherosclerosis. Further, a high ApoB/ApoA-I ratio was specifically associated with frontal domain dysfunction.

## 1. Introduction

Post-stroke cognitive impairment (PSCI) is a significant cause of morbidity and mortality following acute stroke [1,2,3,4]. Cognitive deficits are observed in up to 70% of post-stroke survivors, with variations depending on factors such as the study population, definition of cognitive impairment, assessment methods, and timing of evaluation [5,6]. PSCI can occur after acute ischemic stroke of various etiologies, including small vessel disease, large artery atherosclerosis, and cardioembolism, among others. While several modifiable risk factors, such as characteristics of the index stroke, vascular risk factors, and underlying brain health, contribute to the development of PSCI [7], the identification of biomarkers for predicting PSCI remains an active area of research [8,9,10].

Although dyslipidemia and conventional lipid profiles, including total cholesterol [11], low-density lipoprotein [12], and high-density lipoprotein, have been extensively studied as vascular risk factors, their association with PSCI has yielded inconsistent results in previous cohort studies [13,14,15]. On the other hand, recent research has suggested that specific atherogenic lipoproteins, such as apolipoprotein B (ApoB) and apolipoprotein A-I (ApoA-I), and their ratio may play significant roles in both cognitive function [16] and atherosclerotic stroke [17,18,19]. ApoB is known to generate pro-inflammatory products and exacerbate atherosclerosis in arterial walls [20], while ApoA-I, a major component of high-density lipoprotein cholesterol, exerts anti-inflammatory and antioxidant effects and facilitates the transport of cholesterol from the bloodstream to the liver [21,22]. Hence, the ApoB/ApoA-I ratio may serve as a more robust biomarker for predicting both atherosclerotic stroke [23,24] and subsequent cognitive status compared to other cholesterol measures [25].

Based on these considerations, we hypothesized that a high ApoB/ApoA-I ratio may be associated with worse cognitive outcomes following stroke. Therefore, the primary objective of this study is to investigate the association between the ApoB/ApoA-I ratio and PSCI in patients with acute stroke of large artery atherosclerosis etiology. Additionally, we aim to explore whether the ApoB/ApoA-I ratio is associated with domain-specific cognitive deficits, providing further insights into the underlying mechanisms of PSCI.

## 2. Materials and Methods

### 2.1. Study Design and Population

This study employed a retrospective cross-sectional design based on a prospective stroke registry. Written informed consent was obtained from all participating patients or their legal representatives to use clinical data. The study was approved by the Institutional Review Board of Hallym University Sacred Heart Hospital (IRB No.2021-02-010).

We consecutively enrolled patients with acute ischemic stroke who were hospitalized in our hospital. The inclusion criteria for this study were as follows: (1) consecutive ischemic stroke patients with relevant ischemic lesions on the diffusion-weighted image (DWI) between January 2011 and December 2020, (2) admission within 7 days of symptom onset, (3) large artery atherosclerosis etiology according to the Trial of Org 10,172 in Acute Stroke Treatment (TOAST) criteria [26], (4) no history of statin or other lipid-lowering agent use before the index stroke, and (5) availability of neuropsychological battery results 3 to 6 months after stroke onset. Patients with a pre-stroke diagnosis of dementia or a premorbid modified Rankin scale score of more than two were excluded. Patients with global aphasia, hearing difficulty, poor cooperation, or severe neurological deficits that precluded the administration of the neuropsychological battery were also excluded.

### 2.2. Main Exposure and Covariates

Baseline demographics, including age, sex, body mass index, and education level, were collected from the prospective stroke registry. A history of cardiovascular risk factors such as hypertension, diabetes mellitus, dyslipidemia, atrial fibrillation, smoking status, and prior stroke or transient ischemic attack was recorded. Index stroke characteristics, including initial stroke severity assessed by the National Institutes of Health Stroke Scale (NIHSS) score and stroke etiology according to the TOAST classification, were documented. Fasting blood samples were collected on the day after admission for lipid profile assessment, including apolipoprotein B and apolipoprotein A-I, low-density lipoprotein cholesterol, total cholesterol, as well as other serum biochemical indicators. The ApoB/ApoA-I ratio, the main exposure variable, was divided into quintiles, with Q1 serving as the reference category.

All participants underwent brain magnetic resonance imaging (MRI) using either a 1.5 T or 3 T system based on the year of admission. Lesion laterality, multiplicity, and volume were determined using DWI. Lesion locations were further categorized as cortical, subcortical, or infratentorial. Strategic lesion locations were defined as involvement of the hippocampus, basal ganglia, caudate nucleus, thalamus, inferomedial temporal gyrus, and angular gyrus. The burden of small vessel diseases was evaluated based on the degree of white matter hyperintensities using the modified Fazekas scale [27] and the presence of chronic microbleeds in the lobar or deep locations. Medial temporal lobe atrophy was assessed using Schelten’s scale [28].

### 2.3. Neuropsychological Evaluation and Outcome Variables

All participants underwent the 60 min Korean version of the Vascular Cognitive Impairment Harmonization Standards-Neuropsychological Protocol (K-VCIHS-NP) at 3 to 6 months after stroke onset. The K-VCIHS-NP assessed four cognitive domains: memory, frontal/executive function, language, and visuospatial function. The specific details of each neuropsychological test corresponding to the four cognitive domains are provided in Supplemental Table S1. Additionally, participants completed the Korean versions of the Mini-Mental Status Examination (K-MMSE) [29] and the Montreal Cognitive Assessment (K-MoCA) [30] to evaluate their general cognitive status. All cognitive batteries used in the K-VCIHS-NP, K-MMSE, and K-MoCA were standardized and validated for use in the Republic of Korea. To account for variations in age, sex, and education, raw scores were transformed into z-scores before analysis. The premorbid cognitive status of each participant was assessed using the Korean version of the Informed Questionnaire on Cognitive Decline in the Elderly (IQCODE), with a cutoff value of 3.6, indicating the presence of premorbid cognitive decline [31]. The primary outcome of interest in this study was the development of post-stroke cognitive impairment (PSCI), defined as having a z-score of less than −2 standard deviations in at least one cognitive domain.

### 2.4. Statistical Analysis

Descriptive statistics were used to summarize the data. Continuous variables were reported as means ± standard deviation or medians with interquartile range, depending on their distribution. Categorical variables were presented as numbers and frequencies. Baseline demographic and clinical characteristics were compared between the normal cognition group and the PSCI group using appropriate statistical tests. Student’s *t*-test or the Mann–Whitney U test was used for continuous variables, while the chi-square test or Fisher’s exact test was employed for categorical variables. To examine the differences in clinical characteristics among quintiles of ApoB/ApoA-I, analysis of variance, the Kruskal–Wallis test, or the chi-square test was applied, as appropriate. Univariable and multivariable logistic regression analyses were conducted to investigate the association between the quintiles of ApoB/ApoA-I and PSCI, using Q1 as the reference value. Multivariable models were adjusted for prespecified demographic variables, including age, sex, and education, as well as variables with a *p*-value less than 0.2 in the univariate analysis. Odds ratios (OR) or adjusted odds ratios (aOR), along with their corresponding 95% confidence intervals (CI), were reported to demonstrate the associations. For the secondary analysis, an analysis of covariance (ANCOVA) was performed to compare the z-scores of each cognitive domain and global cognitive functions among quintiles of ApoB/ApoA-I, adjusting for age, sex, and education level. All statistical analyses were carried out using R (version 4.3.3; R Foundation for Statistical Computing), and a two-sided *p*-value less than 0.05 was considered statistically significant.

## 3. Results

**Figure 1:**
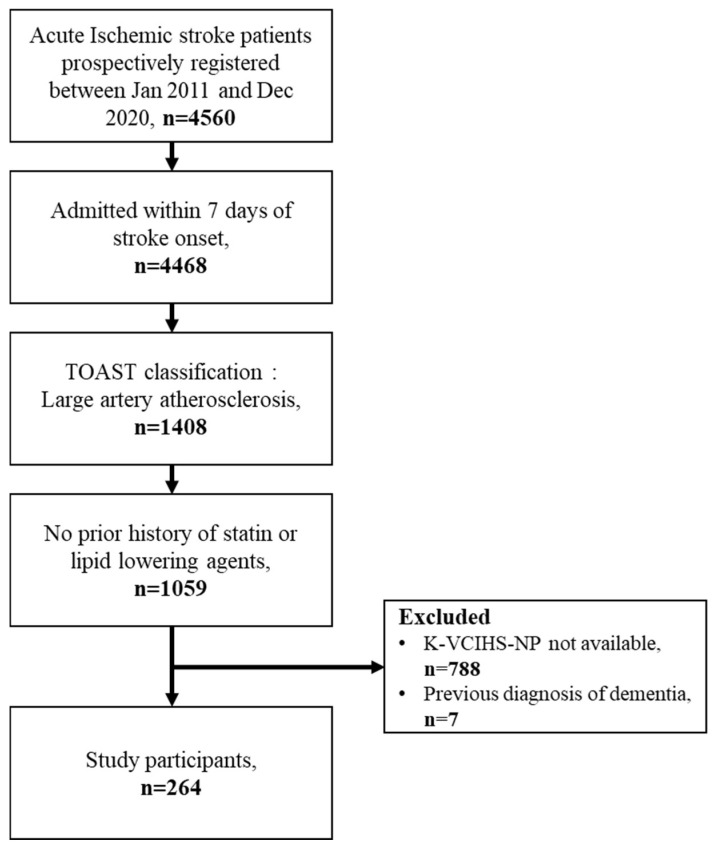
The study flowchart.

Among the 4560 acute ischemic stroke patients assessed during the study period, a total of 1408 patients were identified as having large artery atherosclerosis. Among these patients, 1059 were determined to be naive to statin or other lipid-lowering agents. Finally, after excluding patients with a previous diagnosis of dementia and those who did not undergo the K-VCIHS-NP assessment at 3 to 6 months, 264 patients were included in the analysis (Figure 1). The included patients had a mean age of 65.9 ± 11.6 years. Upon admission, the median NIHSS score was 2 (interquartile range, IQR: 1–5), and the median ApoB/ApoA-I ratio was 0.814 (IQR: 0.762–0.884). Overall, PSCI was observed in 91 out of the 264 patients (34.6%) at the 3 to 6 months after stroke onset.

The baseline characteristics of the study participants according to the ApoB/ApoA-I quintile groups are summarized in Table 1. The only significant differences observed were in the frequency of a history of dyslipidemia and the level of total cholesterol and low-density lipoprotein. No other significant differences were found among the groups (Table 1).

Table 2 presents the differences between the group without cognitive impairment and those who developed PSCI. Participants with PSCI had higher NIHSS scores, larger stroke volume, a higher smoking tendency, and were more likely to have cortical or strategic lesions. On the other hand, they were less likely to have infratentorial lesions. Furthermore, PSCI patients exhibited a higher burden of white matter changes (Table 2).

In the multivariable logistic regression analysis, the highest quintile (Q5) of the ApoB/ApoA-I ratio was found to be significantly associated with the development of PSCI at 3 to 6 months after stroke compared with Q1 (adjusted odds ratio [aOR], 3.00; 95% CI, 1.10–8.20). The adjusted covariates included age, sex, education levels, BMI, systolic blood pressure, previous history of diabetes, smoking, fasting blood sugar, initial stroke severity, stroke volume, presence of cortical, infratentorial or strategic lesions, modified Fazekas scale, and total medial temporal lobe atrophy (MTLA) scores. Among these variables, fasting blood sugar, stroke volume, presence of strategic lesions, and modified Fazekas scale grade 3 were significantly associated with PSCI after adjustment (Table 3).

We also compared the z-scores of each cognitive domain and global cognitive functions among the quintile groups of ApoB/ApoA-I. While the z-scores and raw scores of the K-MMSE and K-MoCA were not significantly different between the groups, the z-scores of the frontal domain showed significant differences and tended to be lower in the Q5 group (Table 4).

## 4. Discussion

The present study revealed several interesting findings. First, among acute ischemic stroke patients with large artery atherosclerosis, a substantial proportion (34.6%) developed PSCI within 3 to 6 months after the stroke. Second, the highest quintile (Q5) of the ApoB/ApoA-I ratio was associated with a three-fold increased risk of developing PSCI compared to the reference group (Q1). Furthermore, the study revealed an association between a higher ApoB/ApoA-I ratio and frontal domain dysfunctions.

The underlying mechanisms that contribute to PSCI are multifaceted and remain incompletely elucidated. However, it is postulated that they encompass a blend of immediate neuronal damage instigated by the stroke alongside more indirect influences such as sustained inflammation, oxidative stress, and vascular dysfunction [15,32]. The ApoB/ApoA-I ratio, a measure of the balance between atherogenic and anti-atherogenic lipoproteins, emerged as a significant predictor of PSCI in our study. The biological plausibility of this association is supported by the known roles of ApoB and ApoA-I in vascular health and inflammation [33,34,35]. Previous research has suggested that ApoB, the primary protein in low-density lipoprotein (LDL), contributes to atherogenesis and inflammation [36], while ApoA-I, a key component of high-density lipoprotein (HDL), exhibits anti-inflammatory and antioxidant properties, and promotes vascular health [37]. Consistent with this understanding, existing literature has underscored that elevated ApoB, diminished ApoA-I, and an increased ApoB/ApoA-I ratio are robust indicators of both cardiovascular diseases [38] and dementia [16] in the general population. Consequently, an elevated ApoB/ApoA-I ratio, which signifies a state of enhanced inflammation, oxidation, and atherogenesis, could potentially augment neuronal damage subsequent to a stroke, thereby precipitating cognitive impairment. Our previous investigations have also demonstrated a connection between the neutrophil-to-lymphocyte ratio, an indirect indicator of systemic inflammation, and the incident PSCI [9]. Additionally, we have established that the state of malnutrition, which is intimately linked with pro-oxidant and pro-inflammatory conditions, plays a role in the deterioration of cognitive functions following a stroke [8]. This study found that the highest quintile of the ApoB/ApoA-I ratio was associated with a three-fold increased risk of developing PSCI, reinforcing the potential role of this lipid imbalance in post-stroke cognitive outcomes.

Moreover, the study unveiled an intriguing association between a higher ApoB/ApoA-I ratio and frontal domain dysfunctions. The frontal lobes of the brain are responsible for executive functions, decision-making, and attentional control, which are critical for daily living activities [39]. This suggests that the vascular damage and inflammation associated with an elevated ApoB/ApoA-I ratio may specifically affect these cognitive functions. Our finding aligns with existing literature that links vascular risk factors with the development of vascular cognitive impairment and vascular dementia, where executive dysfunction is a prominent feature [7,39].

Our study has important clinical implications. The identification of the ApoB/ApoA-I ratio as a significant predictor of PSCI could enable better risk stratification of cognitive impairment for stroke patients and provide a target for interventions to prevent PSCI. Since lifestyle factors and specific medications can influence both ApoB and ApoA-I, interventions aiming at optimizing the ApoB/ApoA-I ratio could potentially reduce the risk of PSCI. This hypothesis needs to be tested in future clinical trials.

This study is subject to several limitations that should be acknowledged. First, the exclusion of acute stroke patients with severe neurological deficits or aphasia who were unable to undergo the neuropsychological battery may limit the generalizability of the findings to the entire acute stroke population. This exclusion could introduce a bias in the study sample, and caution should be exercised when interpreting the results. Further, an additional potential limitation of our study is the homogeneity of our sample, as all subjects were recruited from a single tertiary university hospital. This restricts the generalizability of our findings to broader populations that may be represented in different institutions or geographical locations. Second, the study design was cross-sectional and observational in nature, which prevents the establishment of a causal association between the ApoB/ApoA-I ratio and the development of PSCI. While significant associations were observed, it is important to recognize that causality cannot be inferred from this study alone. Further prospective studies or randomized controlled trials are needed to explore the causal relationship between dyslipidemia, ApoB/ApoA-I ratio, and PSCI. Despite these limitations, this study provides important preliminary evidence regarding the association between the ApoB/ApoA-I ratio and the development of PSCI in patients with large artery atherosclerosis. Future studies addressing these limitations and investigating potential mechanisms underlying this association would contribute to a better understanding of the role of lipid profiles in PCSI.

## 5. Conclusions

In conclusion, our study provides evidence that the ApoB/ApoA-I ratio at the acute stage of ischemic stroke is independently associated with the development of PSCI at 3 to 6 months in patients with large artery atherosclerosis. Specifically, a higher ApoB/ApoA-I ratio is associated with frontal domain dysfunction. These findings have implications for understanding the underlying pathophysiology of PSCI and may potentially impact the management and treatment of cognitive impairment following stroke.

## Figures and Tables

**Table 1:** Demographic and Clinical Characteristics of Patients According to the ApoB/ApoAI Ratio Quintile Groups.

ApoB/ApoA-I Ratio Quintile Groups	Q1 (*n* = 52)	Q2 (*n* = 53)	Q3 (*n* = 52)	Q4 (*n* = 53)	Q5 (*n* = 53)	*p*-Value
PSCI	13 (25.0%)	18 (34.0%)	20 (38.5%)	17 (32.1%)	23 (43.4%)	0.351
ApoB	73.3 ± 13.0	82.0 ± 15.0	100.8 ± 17.0	106.5 ± 15.7	132.2 ± 20.5	<0.001
ApoA1	145.0 ± 24.0	123.4 ± 23.4	127.8 ± 22.6	114.0 ± 17.3	106.8 ± 17.3	<0.001
ApoB/ApoA1 ratio	0.5 ± 0.1	0.7 ± 0.0	0.8 ± 0.0	0.9 ± 0.0	1.3 ± 0.2	<0.001
Demographics						
Age	68.5 ± 12.4	66.9 ± 11.4	63.6 ± 11.2	64.6 ± 10.9	65.8 ± 12.1	0.240
Sex (male)	28 (53.8%)	35 (66.0%)	34 (65.4%)	39 (73.6%)	33 (62.3%)	0.325
Education Level						0.718
0 year	4 (7.7%)	1 (1.9%)	6 (11.5%)	1 (1.9%)	3 (5.7%)	
1–6 years	15 (28.8%)	18 (34.0%)	14 (26.9%)	14 (26.4%)	17 (32.1%)	
6–12 years	22 (42.3%)	26 (49.1%)	22 (42.3%)	29 (54.7%)	24 (45.3%)	
>12 years	11 (21.2%)	8 (15.1%)	10 (19.2%)	9 (17.0%)	9 (17.0%)	
BMI	23.7 ± 3.6	24.2 ± 3.3	24.4 ± 3.5	24.3 ± 3.2	24.1 ± 2.7	0.807
Previous mRS	0.2 ± 0.6	0.2 ± 0.8	0.2 ± 0.6	0.2 ± 0.6	0.1 ± 0.4	0.739
Stroke Characteristics						
Initial NIHSS Stroke Scale	2.0 [1.0;5.0]	2.0 [0.0;5.0]	2.0 [1.0;5.0]	2.0 [1.0;5.0]	3.0 [1.0;6.0]	0.499
Stroke Volume(mm^3^)	11.2 [0.6;95.5]	31.2 [1.9;120.3]	6.6 [0.6;39.2]	4.7 [0.6;70.0]	5.4 [0.5;61.2]	0.202
Thrombolysis						0.494
none	43 (82.7%)	48 (90.6%)	48 (92.3%)	45 (84.9%)	47 (88.7%)	
IVT	6 (11.5%)	5 (9.4%)	4 (7.7%)	7 (13.2%)	5 (9.4%)	
IVT + IAT	3 (5.8%)	0 (0.0%)	0 (0.0%)	1 (1.9%)	1 (1.9%)	
Vascular Risk Factors						
Previous stroke or TIA	4 (7.7%)	6 (11.3%)	6 (11.5%)	6 (11.3%)	4 (7.5%)	0.908
Hypertension	28 (53.8%)	32 (60.4%)	36 (69.2%)	32 (60.4%)	30 (56.6%)	0.570
Diabetes Mellitus	15 (28.8%)	15 (28.3%)	16 (30.8%)	13 (24.5%)	16 (30.2%)	0.961
Hyperlipidemia	5 (9.6%)	9 (17.0%)	17 (32.7%)	18 (34.0%)	22 (41.5%)	0.001
Smoking	15 (28.8%)	22 (41.5%)	21 (40.4%)	20 (37.7%)	27 (50.9%)	0.239
Laboratory Results						
Total Cholesterol	164.4 ± 30.2	167.5 ± 34.4	199.3 ± 50.6	196.0 ± 30.5	227.8 ± 38.1	<0.001
Low-density lipoprotein	92.5 ± 22.0	101.6 ± 23.2	121.0 ± 29.6	131.8 ± 23.0	158.8 ± 29.3	<0.001
Creatinine	1.0 ± 1.7	0.7 ± 0.1	0.8 ± 0.3	0.8 ± 0.2	0.9 ± 0.3	0.503
Hemoglobin	13.6 ± 1.8	14.0 ± 1.3	14.2 ± 1.7	14.0 ± 1.7	14.2 ± 1.5	0.369
Fasting blood sugar	131.0 ± 49.5	121.1 ± 39.6	130.6 ± 52.8	122.8 ± 41.2	123.0 ± 40.4	0.679
Systolic Blood Pressure	148.7 ± 23.6	145.4 ± 23.9	154.8 ± 27.5	150.0 ± 23.7	143.6 ± 25.3	0.173
Lesion Characteristics						
Left Sided Lesion	30 (57.7%)	24 (45.3%)	24 (46.2%)	29 (54.7%)	30 (56.6%)	0.566
Multiples Lesion	7 (13.5%)	4 (7.5%)	4 (7.7%)	4 (7.5%)	4 (7.5%)	0.771
Cortical Lesion	26 (50.0%)	25 (47.2%)	21 (40.4%)	23 (43.4%)	23 (43.4%)	0.881
Subcortical lesion	16 (30.8%)	21 (39.6%)	22 (42.3%)	19 (35.8%)	17 (32.1%)	0.705
Infratentorial Lesion	20 (38.5%)	16 (30.2%)	18 (34.6%)	19 (35.8%)	22 (41.5%)	0.800
Strategic Lesion	16 (30.8%)	16 (30.2%)	17 (32.7%)	21 (39.6%)	12 (22.6%)	0.456
Modified Fazekas Scale						0.929
mFS 0	10 (19.2%)	8 (15.1%)	9 (17.3%)	12 (22.6%)	12 (22.6%)	
mFS 1	23 (44.2%)	25 (47.2%)	26 (50.0%)	21 (39.6%)	26 (49.1%)	
mFS 2	9 (17.3%)	14 (26.4%)	10 (19.2%)	13 (24.5%)	8 (15.1%)	
mFS 3	10 (19.2%)	6 (11.3%)	7 (13.5%)	7 (13.2%)	7 (13.2%)	
Any CMB	13 (25.0%)	10 (18.9%)	8 (15.4%)	9 (16.9%)	8 (15.1%)	0.681
Total MTLA score	2.50 ± 1.83	2.38 ± 1.61	2.31 ± 1.73	1.98 ± 1.72	2.68 ± 1.82	0.326

Abbreviation: ApoB, Apolipoprotein B; ApoAI, Apolipoprotein A-I; PSCI, post-stroke cognitive impairment; BMI, Body mass index; mRS, modified Rankin Scale; NIHSS, National Institute of Health Stroke Scale; IVT, Intravenous thrombolysis; IAT, Intraarterial thrombectomy; TIA, transient ischemic attack; CMB, chronic microbleeds; MTLA, mesial temporal lobe atrophy.

**Table 2:** Demographic and Clinical Characteristics according to the presence of PSCI.

	NCI (*n* = 172)	PSCI (*n* = 92)	*p*-Value
ApoB	96.9 ± 25.2	102.3 ± 28.6	0.124
ApoA1	123.9 ± 24.9	122.3 ± 24.2	0.616
ApoB/ApoA1 ratio	0.81 ± 0.26	0.87 ± 0.31	0.117
Demographics			
Age	65.8 ± 11.9	66.1 ± 11.2	0.838
Sex (male)	108 (62.8%)	62 (67.4%)	0.543
Education Level			0.142
0 year	12 (7.0%)	3 (3.3%)	
1–6 years	49 (28.5%)	30 (32.6%)	
6–12 years	75 (43.6%)	48 (52.2%)	
>12 years	36 (20.9%)	11 (12.0%)	
BMI	24.3 ± 3.1	23.9 ± 3.5	0.281
Previous mRS	0.13 ± 0.54	0.22 ± 0.76	0.314
Stroke Characteristics			
Initial NIHSS Stroke Scale	2.0 [0.5;4.0]	4.0 [1.0;7.0]	<0.001
Stroke Volume(mm^3^)	6.2 [0.81;46.1]	20.7 [0.50;157.3]	0.046
Thrombolysis			0.693
none	151 (87.8%)	80 (87.0%)	
IVT	17 (9.9%)	11 (12.0%)	
IVT + IAT	4 (2.3%)	1 (1.1%)	
Vascular Risk Factors			
Previous stroke or TIA	14 (8.1%)	12 (13.0%)	0.290
Hypertension	102 (59.3%)	57 (62.0%)	0.773
Diabetes Mellitus	43 (25.0%)	32 (34.8%)	0.124
Hyperlipidemia	44 (25.6%)	27 (29.3%)	0.609
Smoking	59 (34.3%)	47 (51.1%)	0.012
Laboratory Results			
Total Cholesterol	187.6 ± 39.0	196.7 ± 51.9	0.140
Low-density lipoprotein	119.2 ± 33.2	124.7 ± 37.5	0.227
Creatinine	0.8 ± 0.2	1.0 ± 1.3	0.302
Hemoglobin	14.0 ± 1.7	14.0 ± 1.6	0.844
Fasting blood sugar	121.8 ± 40.7	132.6 ± 50.9	0.082
Systolic Blood Pressure	150.6 ± 25.4	144.6 ± 23.6	0.064
Lesion Characteristics			
Left Sided Lesion	86 (50.0%)	52 (56.5%)	0.378
Multiple Lesion	16 (9.3%)	7 (7.6%)	0.813
Cortical Lesion	62 (36.0%)	57 (62.0%)	<0.001
Subcortical lesion	60 (34.9%)	35 (38.0%)	0.708
Infratentorial Lesion	77 (44.8%)	18 (19.6%)	<0.001
Strategic Lesion	44 (25.6%)	39 (42.4%)	0.008
Modified Fazekas Scale			0.015
mFS 0	38 (22.1%)	13 (14.1%)	
mFS 1	80 (46.5%)	42 (45.7%)	
mFS 2	38 (22.1%)	16 (17.4%)	
mFS 3	16 (9.3%)	21 (22.8%)	
Any CMB	30 (17.4%)	18 (19.6%)	0.796
Total MTLA score	2.24 ± 1.70	2.61 ± 1.8	0.105

Abbreviation: NCI, no cognitive impairment; PSCI, post-stroke cognitive impairment, ApoB, Apolipoprotein B; ApoAI, Apolipoprotein A-I; BMI, Body mass index; mRS, modified Rankin Scale; NIHSS, National Institute of Health Stroke Scale; IVT, Intravenous thrombolysis; IAT, Intraarterial thrombectomy; TIA, transient ischemic attack; CMB, chronic microbleeds; MTLA, mesial temporal lobe atrophy.

**Table 3:** Multivariable analysis for the predictors of PSCI.

	Crude OR (95% CI)	*p*-Value	Adjusted OR (95% CI)	*p*-Value
ApoB/ApoA1 ratio				
Q1	Reference		Reference	
Q2	1.54 (0.66–3.60	0.316	1.73 (0.61–4.91)	0.303
Q3	1.87 (1.81–4.34)	0.143	2.23 (0.82–6.06)	0.117
Q4	1.42 (0.60–3.32)	0.423	1.56 (0.54–4.47)	0.412
Q5	2.30 (1.00–5.28)	0.049	3.00 (1.10–8.20)	0.032
Age	1.00 (0.98–1.02)	0.837	0.97 (0.94–1.01)	0.172
Sex (male)	1.22 (0.72–2.09)	0.457	0.93 (0.43–2.01)	0.859
BMI	0.95 (0.88–1.04)	0.281	1.07 (0.97–1.18)	0.196
Education years	0.92 (0.67–1.25)	0.587	0.88 (0.54–1.43)	0.614
Systolic Blood Pressure	0.99 (0.98–1.00)	0.066	0.99 (0.98–1.00)	0.099
History of DM	1.60 (0.92–2.77)	0.094	1.49 (0.66–3.36)	0.338
Smoking	2.00 (1.19–3.35)	0.008	1.85 (0.87–3.93)	0.111
Fasting blood sugar	1.01 (1.00–1.01)	0.066	1.01 (1.00–1.02)	0.022
Initial NIHSS	1.13 (1.06–1.21)	<0.001	1.18 (1.00–1.17)	0.057
Stroke Volume (mm^3^)	1.00 (1.00–1.01)	<0.001	1.00 (1.00–1.01)	0.003
Cortical lesions	2.89 (1.71–4.88)	<0.001	1.94 (0.84–4.52)	0.122
Infratentorial lesions	0.30 (0.17–0.54)	<0.001	0.62 (0.24–1.60)	0.321
Strategic lesions	2.14 (1.25–3.66)	0.006	2.49 (1.16–5.38)	0.020
Modified Fazekas Scale				
mFS 0	Reference		Reference	
mFS 1	1.53 (0.74–3.19)	0.252	2.53 (0.98–6.51)	0.055
mFS 2	1.23 (0.52–2.91)	0.636	2.58 (0.80–8.31)	0.112
mFS 3	3.84 (1.55–9.49)	0.004	11.92 (3.05–46.57)	<0.001
Total MTLA score	1.13 (0.97–1.30)	0.106	1.02 (0.83–1.27)	0.831

Adjusted for age, sex, education level, ApoB/ApoAI quintile groups, initial NIHSS, stroke volume(mm^3^) history of diabetes, smoking, fasting blood sugar, systolic blood pressure, cortical lesions, infratentorial lesions, strategic lesions and modified Fazekas scale and Total MTLA scores.

**Table 4:** Domain-specific outcome according to the ApoB/ApoAI ratio using ANCOVA analysis.

ApoB/ApoAI Ratio	Q1 (*n* = 52)	Q2 (*n* = 53)	Q3 (*n* = 52)	Q4 (*n* = 53)	Q5 (*n* = 53)	*p*-Value *
K-MMSE Raw score	25.0 ± 4.9	24.8 ± 5.1	24.7 ± 4.2	25.2 ± 4.8	23.6 ± 4.7	0.689
K-MMSE standardized Z-score	−1.1 ± 1.9	−1.2 ± 2.1	−1.3 ± 1.7	−1.3 ± 2.5	−1.8 ± 1.7	0.414
K-MoCA standardized Z-score	−0.6 ± 1.8	−0.9 ± 1.9	−0.7 ± 1.7	−0.8 ± 2.7	−1.0 ± 1.6	0.689
Frontal domain Z-score	−0.6 ± 0.9	−0.8 ± 1.0	−0.6 ± 1.0	−0.9 ± 0.9	−1.0 ± 1.0	0.045
Language domain Z-score	−0.2 ± 1.2	−0.1 ± 1.2	−0.1 ± 1.0	−0.3 ± 1.2	−0.3 ± 1.2	0.743
Memory domain Z-score	−0.7 ± 1.1	−0.9 ± 1.1	−0.9 ± 1.0	−0.9 ± 1.0	−1.1 ± 1.0	0.311
Visuospatial domain Z-score	−0.8 ± 1.5	−1.3 ± 2.2	−1.4 ± 2.2	−1.2 ± 2.0	−1.3 ± 1.8	0.446

* Adjusted for age, sex, and education years. Abbreviations: K-MMSE, the Korean version of mini-mental status examination; K-MoCA, the Korean version of Montreal cognitive assessment.

## Data Availability

The data presented in this study are available on request from the corresponding author.

## Supplementary Materials

The following supporting information can be downloaded at: https://www.mdpi.com/article/10.3390/nu15214670/s1, Supplemental Table S1. Korean version of the 60 min Vascular Cognitive Impairment Harmonization Standards-Neuropsychology Protocol. References [40,41,42,43,44,45,46,47,48] are cited in the supplementary materials.

## References

[1] Gorelick P.B., Scuteri A., Black S.E., Decarli C., Greenberg S.M., Iadecola C., Launer L.J., Laurent S., Lopez O.L., Nyenhuis D. (2011). Vascular contributions to cognitive impairment and dementia: A statement for healthcare professionals from the american heart association/american stroke association. Stroke.

[2] Leys D., Henon H., Mackowiak-Cordoliani M.A., Pasquier F. (2005). Poststroke dementia. Lancet Neurol..

[3] Pendlebury S.T., Rothwell P.M. (2009). Prevalence, incidence, and factors associated with pre-stroke and post-stroke dementia: A systematic review and meta-analysis. Lancet Neurol..

[4] Sun J.H., Tan L., Yu J.T. (2014). Post-stroke cognitive impairment: Epidemiology, mechanisms and management. Ann. Transl. Med..

[5] Pendlebury S.T., Rothwell P.M., Oxford Vascular S. (2019). Incidence and prevalence of dementia associated with transient ischaemic attack and stroke: Analysis of the population-based Oxford Vascular Study. Lancet Neurol..

[6] Sexton E., McLoughlin A., Williams D.J., Merriman N.A., Donnelly N., Rohde D., Hickey A., Wren M.A., Bennett K. (2019). Systematic review and meta-analysis of the prevalence of cognitive impairment no dementia in the first year post-stroke. Eur. Stroke J..

[7] Lo J.W., Crawford J.D., Desmond D.W., Godefroy O., Jokinen H., Mahinrad S., Bae H.J., Lim J.S., Kohler S., Douven E. (2019). Profile of and risk factors for poststroke cognitive impairment in diverse ethnoregional groups. Neurology.

[8] Lee M., Lim J.S., Kim Y., Lee J.H., Kim C.H., Lee S.H., Jang M.U., Oh M.S., Lee B.C., Yu K.H. (2021). Association between Geriatric Nutritional Risk Index and Post-Stroke Cognitive Outcomes. Nutrients.

[9] Lee M., Lim J.S., Kim C.H., Lee S.H., Kim Y., Hun Lee J., Jang M.U., Sun Oh M., Lee B.C., Yu K.H. (2021). High Neutrophil-Lymphocyte Ratio Predicts Post-stroke Cognitive Impairment in Acute Ischemic Stroke Patients. Front. Neurol..

[10] Lee M., Lim J.S., Kim Y., Lee J.H., Kim C.H., Lee S.H., Jang M.U., Oh M.S., Lee B.C., Yu K.H. (2021). Effects of Glycemic Gap on Post-Stroke Cognitive Impairment in Acute Ischemic Stroke Patients. Brain Sci..

[11] Pascoe M., Ski C.F., Thompson D.R., Linden T. (2019). Serum cholesterol, body mass index and smoking status do not predict long-term cognitive impairment in elderly stroke patients. J. Neurol. Sci..

[12] Kim K.Y., Shin K.Y., Chang K.A. (2022). Potential Biomarkers for Post-Stroke Cognitive Impairment: A Systematic Review and Meta-Analysis. Int. J. Mol. Sci..

[13] Cheng Y., Zhu H., Chen J., Li L., Liu C., Gao Y., Sun D. (2022). Serum TG/HDL-C level at the acute phase of ischemic stroke is associated with post-stroke cognitive impairment. Neurol. Sci..

[14] Dong Y., Ding M., Cui M., Fang M., Gong L., Xu Z., Zhang Y., Wang X., Xu X., Liu X. (2021). Development and validation of a clinical model (DREAM-LDL) for post-stroke cognitive impairment at 6 months. Aging.

[15] Rost N.S., Brodtmann A., Pase M.P., van Veluw S.J., Biffi A., Duering M., Hinman J.D., Dichgans M. (2022). Post-Stroke Cognitive Impairment and Dementia. Circ. Res..

[16] Gong J., Harris K., Peters S.A.E., Woodward M. (2022). Serum lipid traits and the risk of dementia: A cohort study of 254,575 women and 214,891 men in the UK Biobank. eClinicalMedicine.

[17] Li Z., Zhang D., Song Z., Cui X., Liu L., Ding Y., Xue J., Zhang X., Ma R., Zhu X. (2023). Elevated ApoB/ApoA-Iota ratio is associated with poor outcome in acute ischemic stroke. J. Clin. Neurosci..

[18] Liu D., Zhang Y., Wang C., Zuo H. (2022). Association of the ApoB/ApoA-I ratio with stroke risk: Findings from the China Health and Nutrition Survey (CHNS). Nutr. Metab. Cardiovasc. Dis..

[19] Park J.H., Hong K.S., Lee E.J., Lee J., Kim D.E. (2011). High levels of apolipoprotein B/AI ratio are associated with intracranial atherosclerotic stenosis. Stroke.

[20] Sniderman A.D., Faraj M. (2007). Apolipoprotein B, apolipoprotein A-I, insulin resistance and the metabolic syndrome. Curr. Opin. Lipidol..

[21] Bielicki J.K., Oda M.N. (2002). Apolipoprotein A-I(Milano) and apolipoprotein A-I(Paris) exhibit an antioxidant activity distinct from that of wild-type apolipoprotein A-I. Biochemistry.

[22] Nissen S.E., Tsunoda T., Tuzcu E.M., Schoenhagen P., Cooper C.J., Yasin M., Eaton G.M., Lauer M.A., Sheldon W.S., Grines C.L. (2003). Effect of recombinant ApoA-I Milano on coronary atherosclerosis in patients with acute coronary syndromes: A randomized controlled trial. JAMA.

[23] Fahmy E.M., El Awady M.A.E.S., Sharaf S.A.-A., Selim N.M., Abdo H.E.S., Mohammed S.S. (2020). Apolipoproteins A1 and B and their ratio in acute ischemic stroke patients with intracranial and extracranial arterial stenosis: An Egyptian study. Egypt. J. Neurol. Psychiatry Neurosurg..

[24] Kostapanos M.S., Christogiannis L.G., Bika E., Bairaktari E.T., Goudevenos J.A., Elisaf M.S., Milionis H.J. (2010). Apolipoprotein B-to-A1 ratio as a predictor of acute ischemic nonembolic stroke in elderly subjects. J. Stroke Cerebrovasc. Dis..

[25] Walldius G., Jungner I. (2006). The apoB/apoA-I ratio: A strong, new risk factor for cardiovascular disease and a target for lipid-lowering therapy—A review of the evidence. J. Intern. Med..

[26] Adams H.P., Bendixen B.H., Kappelle L.J., Biller J., Love B.B., Gordon D.L., Marsh E.E. (1993). Classification of subtype of acute ischemic stroke. Definitions for use in a multicenter clinical trial. TOAST. Trial of Org 10172 in Acute Stroke Treatment. Stroke.

[27] Fazekas F., Kleinert R., Offenbacher H., Schmidt R., Kleinert G., Payer F., Radner H., Lechner H. (1993). Pathologic correlates of incidental MRI white matter signal hyperintensities. Neurology.

[28] Scheltens P., Leys D., Barkhof F., Huglo D., Weinstein H.C., Vermersch P., Kuiper M., Steinling M., Wolters E.C., Valk J. (1992). Atrophy of medial temporal lobes on MRI in “probable” Alzheimer’s disease and normal ageing: Diagnostic value and neuropsychological correlates. J. Neurol. Neurosurg. Psychiatry.

[29] Kang Y., Na D.L., Hahn S. (1997). A validity study on the korean mini-mental state examination (K-MMSE) in dementia patients. J. Korean Neurol. Assoc..

[30] Kang Y., Park J.S., Yu K.H., Lee B.C. (2009). A Reliability, Validity, and Normative Study of the Korean-Montreal Cognitive Assessment(K-MoCA) as an Instrument for Screening of Vascular Cognitive Impairment(VCI). Korean J. Clin. Psychol..

[31] Park M.H. (2017). Informant questionnaire on cognitive decline in the elderly (IQCODE) for classifying cognitive dysfunction as cognitively normal, mild cognitive impairment, and dementia. Int. Psychogeriatr..

[32] Tian Z., Ji X., Liu J. (2022). Neuroinflammation in Vascular Cognitive Impairment and Dementia: Current Evidence, Advances, and Prospects. Int. J. Mol. Sci..

[33] Sniderman A.D., St-Pierre A.C., Cantin B., Dagenais G.R., Despres J.P., Lamarche B. (2003). Concordance/discordance between plasma apolipoprotein B levels and the cholesterol indexes of atherosclerotic risk. Am. J. Cardiol..

[34] Rosenson R.S., Brewer H.B., Ansell B.J., Barter P., Chapman M.J., Heinecke J.W., Kontush A., Tall A.R., Webb N.R. (2016). Dysfunctional HDL and atherosclerotic cardiovascular disease. Nat. Rev. Cardiol..

[35] Walldius G., Jungner I., Holme I., Aastveit A.H., Kolar W., Steiner E. (2001). High apolipoprotein B, low apolipoprotein A-I, and improvement in the prediction of fatal myocardial infarction (AMORIS study): A prospective study. Lancet.

[36] Faraj M., Messier L., Bastard J.P., Tardif A., Godbout A., Prud’homme D., Rabasa-Lhoret R. (2006). Apolipoprotein B: A predictor of inflammatory status in postmenopausal overweight and obese women. Diabetologia.

[37] Umemoto T., Han C.Y., Mitra P., Averill M.M., Tang C., Goodspeed L., Omer M., Subramanian S., Wang S., Den Hartigh L.J. (2013). Apolipoprotein AI and high-density lipoprotein have anti-inflammatory effects on adipocytes via cholesterol transporters: ATP-binding cassette A-1, ATP-binding cassette G-1, and scavenger receptor B-1. Circ. Res..

[38] Carnevale Schianca G.P., Pedrazzoli R., Onolfo S., Colli E., Cornetti E., Bergamasco L., Fra G.P., Bartoli E. (2011). ApoB/apoA-I ratio is better than LDL-C in detecting cardiovascular risk. Nutr. Metab. Cardiovasc. Dis..

[39] Sachdev P.S., Brodaty H., Valenzuela M.J., Lorentz L., Looi J.C., Wen W., Zagami A.S. (2004). The neuropsychological profile of vascular cognitive impairment in stroke and TIA patients. Neurology.

[40] Yu K.H., Cho S.J., Oh M.S., Jung S., Lee J.H., Shin J.H., Koh I.-S., Cha J.-K., Park J.-M., Bae H.-J. (2013). Cognitive impairment evaluated with vascular cognitive impairment harmonization standards in a multicenter prospective stroke cohort in Korea. Stroke.

[41] Kang Y.W., Chin J.H., Na D.L., Lee J.H., Park J.S. (2000). A normative study of the Korean version of Controlled Oral Word Association Test (COWAT) in the elderly. Korean J. Clin. Psychol..

[42] Yum T.H., Park Y.S., Oh K.J., Kim J.H., Lee Y.H. (1992). Manual for Korean-Wechsler Adult Intelligence Scale.

[43] Yi H., Chin J.H., Lee B.H., Kang Y., Na D.L. (2007). Development and validation of Korean version of trail making test for elderly persons. Dement. Neurocogn. Disord..

[44] Kang Y., Kim H.H., Na D.L. (1999). A short form of the Korean-Boston Naming Test (K-BNT) for using in dementia patients. Korean J. Clin. Psych..

[45] Kang Y., Na D.L. (2003). Seoul Neuropsychological Screening Battery.

[46] Lee D.W., Lee J.Y., Ryu S.G., Cho S.J., Hong C.H., Lee J.H., Choi Y.M., Kim B.S., Park E.J., Park S.H. (2005). Validity of the Korean version of Informant Questionnaire on Cognitive Decline in the Elderly(IQCODE). J. Korean Geriatr. Soc..

[47] Kang Y. (2006). A normative study of the Korean-Mini Mental State Examination(K-MMSE) in the elderly. Korean J. Psych..

[48] Kang S.J., Choi S.H., Lee B.H., Kwon J.C., Na D.L., Han S.H., Korean Dementia Research Group (2002). The reliability and validity of the Korean Instrumental Activities of Daily Living (K-IADL). J. Korean Neurol. Assoc..

