# Supplementary material for: High ApoB/ApoA-I Ratio Predicts Post-Stroke Cognitive Impairment in Acute Ischemic Stroke Patients with Large Artery Atherosclerosis
Source: Nutrients. 2023 Nov 4;15(21):4670. doi: 10.3390/nu15214670 (PMC10648714; doi:10.3390/nu15214670)
Supplement: Supplementary file 1 [file nutrients-15-04670-s001.zip › nutrients-2685139-supplementary.pdf]

Supplemental Table S1. Korean version of the 60-minute Vascular Cognitive Impairment Harmonization Standards-Neuropsychology Protocol[1]

Cognitive Domains	Korean Version of VCIHS-NP protocol
Executive/Activation	Animal Naming (semantic fluency) [2]<br>Korean Controlled Oral Word Association Test (ㄱ, ㅇ, ㅅ) [2]<br>Digit Symbol-Coding[3]<br>Korean-Trail making Test-Elderly's version[4]
Language	Korean-Boston Naming test: Short form A[5]
Visuospatial	Rey Complex Figure Test Copy[6]
Memory	Seoul Verbal Learning Test[6]
Others	Informant Questionnaire of Cognitive Decline in the Elderly (IQCODE) [7]
	Korean-Mini-Mental State Examination[8]
	Korean-Instrumental Activity of Daily Living[9]

References

1. Yu KH, Cho SJ, Oh MS, Jung S, Lee JH, Shin JH, et al. Cognitive impairment evaluated with vascular cognitive impairment harmonization standards in a multicenter prospective stroke cohort in Korea. Stroke. 2013;44:786-788

2. Kang YW, Chin JH, Na DL, Lee JH, Park JS. A normative study of the Korean version of Controlled Oral Word Association Test (COWAT) in the elderly. Korean J Clin Psychol. 2000;19:385-392

3. Yum TH, Park YS, Oh KJ, Kim JH, Lee YH. Manual for Korean-Wechsler Adult Intelligence Scale. Seoul: Korea Guidance; 1992.

4. Yi H, Chin JH, Lee BH, Kang Y, Na DL. Development and validation of Korean version of trail making test for elderly persons. Dement Neurocognitive Disord. 2007;6:54-66

5. Kang Y, Kim HH, Na DL. A short form of the Korean-Boston Naming Test (K-BNT) for using in dementia patients. Korean J Clin Psych. 1999;18:125-138

6. Kang Y, Na DL. Professional manual; Seoul neuropsychological screening battery. Seoul: Human brain research and consulting; 2003.

7. Lee DW, Lee JY, Ryu SG, Cho SJ, Hong CH, Lee JH, et al. Validity of the Korean version of Informant Questionnaire on Cognitive Decline in the Elderly(IQCODE). J Korean Geriatr Soc. 2005;9:196-204

8. Kang Y. A normative study of the Korean-Mini Mental State Examination(K-MMSE) in the elderly. Korean J Psych. 2006;25:1-12

9. Kang SJ, Choi SH, Lee BH, Kwon JC, Na DL, Han SH, Korean Dementia Research Group. The reliability and validity of the Korean Instrumental Activities of Daily Living (K-IADL). J Korean Neurol Assoc 2002;20:8~14
